# RE-EVALUATING FORTITUDE, VULNERABILITY, AND FRAILTY IN CARE HOME STORIES

**DOI:** 10.1093/geroni/igae098.1443

**Published:** 2024-12-31

**Authors:** Ulla Kriebernegg

**Affiliations:** University of Graz, Graz, Steiermark, Austria

## Abstract

This paper analyzes how concepts of fortitude, vulnerability, frailty, and care are linked from the perspective of literary gerontology. Discussing the newly emerging genre of the care home novel, the paper shows how a close reading can challenge traditional assumptions about bodies on the margins of life and death as merely existing in the “black hole” of the fourth age (Gilleard and Higgs 2010). Examples are drawn from stories that are set in long-term care facilities that resemble “total institutions” (Goffman 1961), such as May Sarton’s As We Are Now (1973) or Oscar Casares’s Amigoland (2009), where aging into old age is often narrated as a creative process that allows for agency and involves the renegotiation of one’s identity despite extreme vulnerability. The humanities lens here examines such novels as “Reifungsromane” (novels of ripening, Waxman 1990) or “Vollendungsromane” (novels of completion, Rooke 1992), where vulnerability is represented as a meaningful condition that facilitates resistance in the face of loss and precarity. As such, these are striking individual narratives of fortitude and self-determination that are less about “successful aging,” than about “successful frailty,” which Wendy Lustbader describes as “turning the ‘nothing’ of empty time into the ‘something’ of good days” (1991: 14). Conclusions reiterate how vulnerability is a shared ontological condition, and why a humanities perspective is important to highlight vulnerability as a current socio-political issue in connection with care ethics and social justice.

